# Engineering of a Peptide α‐N‐Methyltransferase to Methylate Non‐Proteinogenic Amino Acids

**DOI:** 10.1002/anie.202100818

**Published:** 2021-05-17

**Authors:** Haigang Song, Antony J. Burton, Sally L. Shirran, Jūratė Fahrig‐Kamarauskaitė, Hannelore Kaspar, Tom W. Muir, Markus Künzler, James H. Naismith

**Affiliations:** ^1^ Division of Structural Biology Wellcome Centre for Human Genetics Roosevelt Drive Oxford OX3 7BN UK; ^2^ The Research Complex at Harwell Harwell Campus Oxford OX11 0FA UK; ^3^ The Rosalind Franklin Institute Harwell Campus Oxford OX11 0FA UK; ^4^ Department of Chemistry Frick Chemistry Laboratory Princeton University Princeton NJ USA; ^5^ Biomedical Sciences Research Complex, North Haugh University of St. Andrews Fife KY16 9ST UK; ^6^ Department of Biology Institute of Microbiology Eidgenössische Technische Hochschule (ETH) Zürich Zürich Switzerland

**Keywords:** cyclic peptide, non-proteinogenic amino acids, RiPPs, split intein, α-N-methylation

## Abstract

Introduction of α‐N‐methylated non‐proteinogenic amino acids into peptides can improve their biological activities, membrane permeability and proteolytic stability. This is commonly achieved, in nature and in the lab, by assembling pre‐methylated amino acids. The more appealing route of methylating amide bonds is challenging. Biology has evolved an α‐N‐automethylating enzyme, OphMA, which acts on the amide bonds of peptides fused to its C‐terminus. Due to the ribosomal biosynthesis of its substrate, the activity of this enzyme towards peptides with non‐proteinogenic amino acids has not been addressed. An engineered OphMA, intein‐mediated protein ligation and solid‐phase peptide synthesis have allowed us to demonstrate the methylation of amide bonds in the context of non‐natural amides. This approach may have application in the biotechnological production of therapeutic peptides.

## Graphical abstract

Peptide backbone methylation is a highly desired modification. Using split intein, synthetic peptides and an engineered variant of the methylase, fOphMA2, it was possible to enzymatically methylate amide bonds involving non‐natural amino acids. This has application in the biotechnological production of therapeutic peptides.

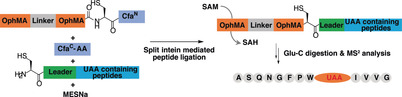

Peptide cyclization, backbone N‐methylation, and the use of d and non‐proteinogenic amino acids are common strategies in medicinal chemistry to enhance the metabolic stability, cell permeability, and bioavailability of synthetic peptides.[^1^, ^2^, ^3^, ^4^, ^5^, ^6^] Methylation is a particularly desirable modification since it reduces the polarity of the amide bond, introduces conformational constraints and sequential introduction appears additive in terms of increasing membrane permeability.[^6^, ^7^, ^8^] Cyclosporine A, the widely prescribed immunosuppressant, exemplifies this with its seven backbone N‐methylations, an aminobutyric acid (Abu), N‐methylated butenyl‐threonine (Bmt) and d‐alanine residues.^[9]^ Cyclosporin A is a non‐ribosomal peptide natural product (NRPs) where methylation is carried out on the building blocks by methyltransferase domains prior to peptide bond formation.[^10^, ^11^] Manipulating NRP synthetases to generate novel N‐methylated unnatural amino acids is challenging due to the complexity of the assembly lines.[^12^, ^13^] N‐methylated amino acids have been incorporated into peptides by expanding or reprogramming the genetic code and by using pre‐charged N‐methyl aminoacyl‐tRNAs in cell‐free ribosomal translation systems.[^14^, ^15^, ^16^, ^17^, ^18^] Traditionally this approach had low efficiency but the novel flexizyme system has been able to produce polymethylated peptides using tRNA loaded with methylated amino acid building blocks.[^17^, ^18^, ^19^, ^20^, ^21^, ^22^] These approaches do however not methylate the peptide bond itself and, thus, do not utilize the full diversity available through chemical peptide synthesis. Enzymatic methylation of the amide bond has recently been discovered[^23^, ^24^] (Figure 1 a), but its potential for processing amide bonds involving non‐proteogenic amino acids has not been explored. The enzymatic methylation of chemically synthesized peptides creates the possibility of highly diverse molecules.

**Figure 1:**
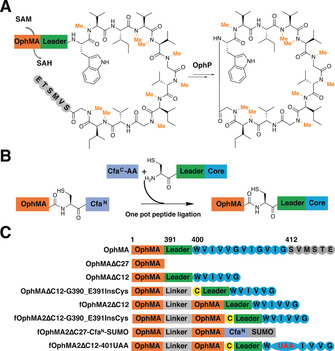
OphMA‐ and split‐intein‐mediated peptide ligation. A) OphMA catalyzes nine processive backbone N‐methylations of its C‐terminal peptide which is converted to omphalotin A by OphP (macrocyclase).[^23^, ^24^, ^25^] The leader peptide is shown green, the follower as gray circles. B) The scheme for one‐pot core peptide (blue) ligation to OphMA mediated by split intein Cfa^N^ and mutated Cfa^C^ (denoted Cfa^C^‐AA) inserts a cysteine at the ligation site (see also Figure S1). C) Key to constructs. Native OphMA (numbers show the first residue of each region); OphMAΔC27 and OphMAΔC12 (27 or 12 C‐terminal residues deleted); OphMAΔC12‐G390_E391insCys as OphMAΔC12 but cysteine (yellow) inserted between 390 and 391; fOphMA2ΔC12 is the fused dimer with 12 C‐terminal residues deleted; fOphMA2ΔC12‐G390_E391InsCys is fOphMA2ΔC12 with cysteine insertion; fOphMA2ΔC27‐Cfa^N^‐SUMO intein and sumo added to C‐terminus; fOphMA2ΔC12‐401UAA the post ligation protein.

Structural analysis of OphMA revealed that the substrate peptide is mainly recognized via hydrogen bonds to substrate backbone carbonyls[^26^, ^27^, ^28^] with side chains of substrate playing a minor role. OphMA has been shown to prefer hydrophobic residues (Gly, Ala, Leu, Ile), small hydrophilic residues (Thr, Ser), tolerate Phe and (albeit inefficiently) methylate Glu.[^23^, ^24^, ^29^] OphMA homologs from other fungi are able to methylate different length core peptides and process His, Cys, Tyr, Gln, and Asp residues.[^28^, ^30^] Since no *in trans* activity of OphMA has been identified,[^27^, ^31^] the substrate scope of these methyltransferases is limited to the 20 proteinogenic amino acids. Genetic code engineering could introduce non‐natural amino acids into OphMA core peptide but limits diversity.[^32^, ^33^, ^34^, ^35^] We therefore chose to develop a system that combined peptide synthesis with enzymatic amide methylation. We selected expressed protein ligation (EPL)[^36^, ^37^] to couple a synthetic peptide to the enzyme using the Cfa split intein system (consensus DnaE intein sequence) as it has excellent thermal and chaotropic stability (Figure 1 b, Figure S1).[^37^, ^38^, ^39^, ^40^, ^41^, ^42^, ^43^]

OphMA functions as an interlocked dimer forming a catenane arrangement with C‐terminal substrate of one monomer folded into the active tunnel of the other monomer.^[26]^ The catenane arrangement of OphMA seems certain to be functionally required. Guided by this structural insight, we truncated OphMA after Gly390 in order to preserve the last α‐helix (S380–E388, thus predicted structural stability), yielding OphMAΔC27 to which the Cfa intein would be added (Figure 1 c). Ligation of peptides to this system would result in a cysteine insertion between residues 390 and 391 of OphMAΔC12 (Figure 1 c). Construction of fused dimers has been shown to improve the expression level, stability and activity of some enzymes.[^44^, ^45^, ^46^] Again influenced by the structure, we constructed a fused OphMA dimer (fOphMA2) (Figure 1 c). The fused dimer consists of Met1 to Asn377 of one monomer linked by (GGGGS)_6_ to the second (variable) monomer.

The C‐terminal peptide of OphMA is insoluble, therefore a soluble test sequence **WVIVVG** was chosen for the core peptide. OphMAΔC12, OphMAΔC12‐G390_E391insCys and fOphMA2ΔC12‐G390_E391insCys (Figure 1 c) were expressed in *E. coli* to evaluate enzyme activity on the test sequence made during ribosomal synthesis.[^26^, ^29^] OphMAΔC12 was purified as a mixture with zero, one and two methylations (Figure 2 a) confirming it was active. Cysteine insertions showed improved methylation when compared to OphMAΔC12 with more mono‐ and di‐methylation. The fused dimer was purified as a well‐folded methylated protein (Figure S2) with improved thermal stability (Figure S3). Both intact mass and MS^2^ analysis identified that Val401 and Val403 were methylated (expected from previous work (Figure S7 & S8)). All three constructs showed the expected methylation pattern for this test sequence; the two C‐terminal residues are methylated.[^23^, ^24^, ^26^, ^27^, ^28^, ^29^] Since the engineered enzymes were active, we proceeded to ligate a peptide containing the test sequence (CEEASQNGFP‐**WVIVVG**, peptide **1,** Table S1).

**Figure 2:**
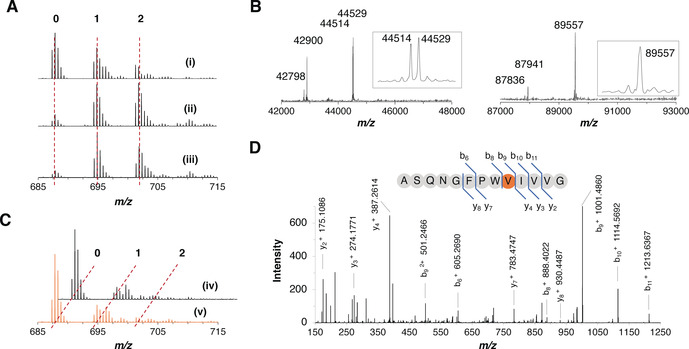
OphMA catalyzes backbone N‐methylation of ligated synthetic peptides. A) MS analysis of C‐terminal peptide of the expressed OphMAΔC12 (i), OphMAΔC12‐G390_E391insCys (ii) and fOphMA2ΔC12‐G390_E391insCys (iii). B) Intact mass analysis of ligated products, OphMAΔC12‐401Val (left) and fOphMA2ΔC12‐401Val (right). C) MS analysis of C‐terminal peptide confirmed N‐methylation for OphMAΔC12‐401Val (iv) and fOphMA2ΔC12‐401Val (v). Both are ligated protein with cysteine insertion between Gly390 and Glu391, and incubated with SAM for three days. D) MS^2^ spectrum confirms the N‐methylated site of fOphMA2ΔC12‐401Val.

Split‐intein fusion variants were constructed with a C‐terminal SUMO fusion tag (simplifying monitoring) for the monomer (OphMAΔC27‐Cfa^N^‐SUMO) and fused dimer (fOphMA2ΔC27‐Cfa^N^‐SUMO, Figure 1 c). Both proteins were purified from *E. coli*[^26^, ^29^] and showed 60 % ligation efficiency in 4 h and reached around 80 % within 20 h (estimated by SDS‐PAGE gels, Figure S4).

ESI‐TOF MS determined the molecular mass as 44 514 Da for OphMAΔC12‐401Val (a peak at 44 529 Da corresponds to oxidation) and 89 557 Da for fOphMA2ΔC12‐401Val, the expected masses for ligated proteins (Figure 2 b, S6). Peaks at 42 900 Da in OphMA and 87 944 Da in fOphMA2 corresponded to unligated product, likely a result of thioester hydrolysis or cleavage. Both OphMAΔC12‐401Val and fOphMA2ΔC12‐401Val were incubated with SAM for three days as previously described[^26^, ^30^] and analysis of Glu‐C‐digested C‐terminal peptides returned twenty percent of the mono‐methylated species (Figure 2 c, S7). MS^2^ confirmed that Val401 was methylated in both proteins, and the di‐methylated species was also detected for fOphMA2 (Figure 2 c, S8) suggesting it was more active. The in vitro activities of the ligated variants were lower than their translated counterparts but validated the approach. Since the fused protein was more stable, readily ligated and more active than monomer we pursued it.

A panel of synthetic peptides with a range of non‐proteinogenic amino acids at position 401 were ligated to fOphMA2ΔC27‐Cfa^N^‐SUMO and the extent of methylation both at the unnatural amino acid and elsewhere in the core peptide judged by mass spectrometry are reported in Table S1, Figure 3 and S6, S7, S8. Interestingly, both Nva and Nle were more efficiently methylated (by around 4‐fold) than valine. The bioorthogonal reactive alkyne containing Pra was also well tolerated. MS^2^ analysis confirmed that in addition to methylation at Val401Pra there was additional methylation on Ile402 or Val403 (Figure S7, S8). Introduction of larger non‐aromatic cyclic hydrophobic side chains reduced methylation with 6 % mono‐methylation at Val401Cha, 11 % mono‐methylation at Val401Chg and 21 % mono‐methylation at Val401Cpg (Figure 3, Table S1, Figure S7, S8). OphMA has previously been shown to methylate phenylalanine in different positions albeit with reduced efficiency.^[29]^ MS and MS^2^ spectra showed that fOphMA2 methylated various aromatic amino acids ranging from 7 % methylation of 4‐F‐Phe to 40 % with 2‐Pal (Table S1, Figure 3 and S7, S8). Detectable methylation at Val401Phg was also seen (Table S1 and Figure S7, S8). Very large side chains (4‐Cl‐Phe, 4‐I‐Phe, 4‐NO_2_‐Phe) prevented methylation. Homoserine was well tolerated and allowed a small amount of additional methylation (Ile402 or Val403, Table S1, Figure S7, S8). The highly branched penicillamine side chain was comparably methylated to valine (Table S1, Figure 3 and S7, S8). The positively charged diaminobutyric acid (Dab) yielded mono‐methylated protein, a surprise since arginine blocks methylation in native OphMA. MS^2^ showed two mono‐methylated species, one with methylated Dab and the other with methylated Ile402 (Figure S8). Residues Aib, d‐Ala, d‐Val and d‐Thr all completely abolished methylation. A variety of non‐α amino acids were tested with only small amounts of mono‐methylation observed for protein with Ahx or Gaba but MS^2^ suggested that it was Ile402 or Val403 which were methylated (Figure S7, S8). We attribute the lack of ligation product with peptide **30** (CEEASQNGFP‐**WVIVVGVIG**) to the peptide's insolubility (Figure S6).

**Figure 3:**
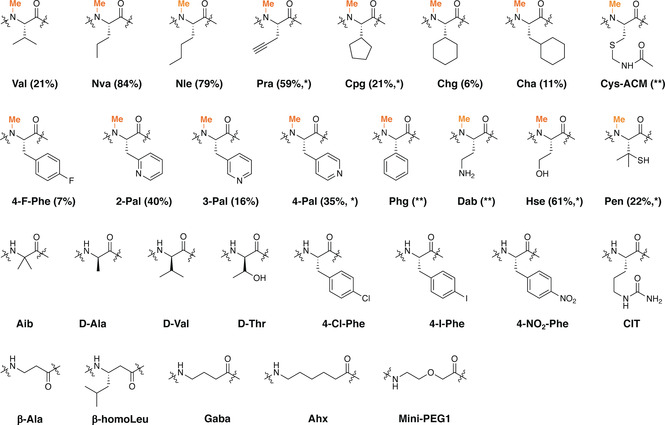
Non‐proteinogenic amino acids N‐methylated by OphMA. The methylation was determined by MS^2^ of the Glu‐C digested fOphMA2‐ligated protein. Values in the bracket represent relative percentage of mono‐methylation species with non‐proteinogenic site methylated and are calculated by integrating all methylated species observed. * Di‐methylation was also detected; ** indicates methylation of the residue but low abundance.

Since we have ligated the peptide to an already folded protein, the residues in the peptide must enter the active site. This was a surprising finding since it requires the enzyme to undergo a large conformational change. The structure had implied substrate peptide entered the active site during the folding.^[29]^ In fact, the enzyme must undergo a conformational change in the active site. The ability to undergo this change without unfolding may explain why a fused dimeric enzyme behaved better. Our data showed the enzyme fOphMA2 was able to process hydrophobic side chains including aromatics but with a preference for smaller unbranched side chains. Despite the mismatch in polarity, we observed the methylation of a small positively charged residue, suggesting small size followed by hydrophobicity dominate substrate tolerance. Structural data had suggested d‐configured amino acids would clash at the active site and indeed none were tolerated. The inefficiency of methylation of Phg suggested that backbone flexibility is important, the first experimental evidence to support the flip and translate model for OphMA hypermethylation.[^26^, ^28^] The lack of activity on non‐α amino acids is likely due to disruption of the critical hydrogen bonding interactions between the substrate peptide and enzyme.[^26^, ^27^, ^28^]

This is the first report of enzymatic N‐methylation of amide bonds involving non‐natural amino acids. Moreover, the fused protein appears to be a useful tool for further exploration, since it was able to process substrates that are added after the enzyme has folded. The fused enzyme is more stable and appears to be more active. Refinement of this strategy could provide access to entirely novel multiply backbone N‐methylated peptides.

## Conflict of interest

M. Künzler is co‐inventor of patent WO2017174760A1.

## Supporting information

As a service to our authors and readers, this journal provides supporting information supplied by the authors. Such materials are peer reviewed and may be re‐organized for online delivery, but are not copy‐edited or typeset. Technical support issues arising from supporting information (other than missing files) should be addressed to the authors.

Supplementary

## References

[1] A. T. Bockus , J. A. Schwochert , C. R. Pye , C. E. Townsend , V. Sok , M. A. Bednarek , R. S. Lokey , J. Med. Chem. 2015, 58, 7409–7418. 26308180 10.1021/acs.jmedchem.5b00919

[2] J. L. Hickey , S. Zaretsky , M. A. St Denis , S. K. Chakka , M. M. Morshed , C. C. G. Scully , A. L. Roughton , A. K. Yudin , J. Med. Chem. 2016, 59, 5368–5376. 27120576 10.1021/acs.jmedchem.6b00222

[3] A. Furukawa , C. E. Townsend , J. Schwochert , C. R. Pye , M. A. Bednarek , R. S. Lokey , J. Med. Chem. 2016, 59, 9503–9512. 27690434 10.1021/acs.jmedchem.6b01246

[4] R. Jwad , D. Weissberger , L. Hunter , Chem. Rev. 2020, 120, 9743–9789. 32786420 10.1021/acs.chemrev.0c00013

[5] T. Rezai , B. Yu , G. L. Millhauser , M. P. Jacobson , R. S. Lokey , J. Am. Chem. Soc. 2006, 128, 2510–2511. 16492015 10.1021/ja0563455

[6] J. Chatterjee , C. Gilon , A. Hoffman , H. Kessler , Acc. Chem. Res. 2008, 41, 1331–1342. 18636716 10.1021/ar8000603

[7] J. Chatterjee , F. Rechenmacher , H. Kessler , Angew. Chem. Int. Ed. 2013, 52, 254–269; 10.1002/anie.201205674 23161799

[8] A. F. B. Räder , F. Reichart , M. Weinmüller , H. Kessler , Bioorg. Med. Chem. 2018, 26, 2766–2773. 28886995 10.1016/j.bmc.2017.08.031

[9] M. Dreyfuss , E. Härri , H. Hofmann , H. Kobel , W. Pache , H. Tscherter , Eur. J. Appl. Microbiol. 1976, 3, 125–133.

[10] A. Lawen , R. Zocher , J. Biol. Chem. 1990, 265, 11355–11360. 2358465

[11] R. D. Süssmuth , A. Mainz , Angew. Chem. Int. Ed. 2017, 56, 3770–3821; 10.1002/anie.201609079 28323366

[12] R. H. Baltz , Nat. Biotechnol. 2006, 24, 1533–1540. 17160059 10.1038/nbt1265

[13] M. A. Fischbach , C. T. Walsh , Chem. Rev. 2006, 106, 3468–3496. 16895337 10.1021/cr0503097

[14] C. J. Noren , S. J. Anthony-Cahill , M. C. Griffith , P. G. Schultz , Science 1989, 244, 182–188. 2649980 10.1126/science.2649980

[15] J. D. Bain , E. S. Diala , C. G. Glabe , T. A. Dix , A. R. Chamberlin , J. Am. Chem. Soc. 1989, 111, 8013–8014.

[16] Y. Shimizu , A. Inoue , Y. Tomari , T. Suzuki , T. Yokogawa , K. Nishikawa , T. Ueda , Nat. Biotechnol. 2001, 19, 751–755. 11479568 10.1038/90802

[17] T. Kawakami , H. Murakami , H. Suga , Chem. Biol. 2008, 15, 32–42. 18215771 10.1016/j.chembiol.2007.12.008

[18] A. O. Subtelny , M. C. T. Hartman , J. W. Szostak , J. Am. Chem. Soc. 2008, 130, 6131–6136. 18402453 10.1021/ja710016v PMC2728122

[19] H. Murakami , A. Ohta , H. Ashigai , H. Suga , Nat. Methods 2006, 3, 357–359. 16628205 10.1038/nmeth877

[20] Y. Goto , T. Katoh , H. Suga , Nat. Protoc. 2011, 6, 779–790. 21637198 10.1038/nprot.2011.331

[21] T. Passioura , H. Suga , Chem. Commun. 2017, 53, 1931–1940. 10.1039/c6cc06951g 28091672

[22] C. Tsiamantas , J. M. Rogers , H. Suga , Chem. Commun. 2020, 56, 4265–4272. 10.1039/d0cc01291b 32267262

[23] N. S. van der Velden , N. Kälin , M. J. Helf , J. Piel , M. F. Freeman , M. Künzler , Nat. Chem. Biol. 2017, 13, 833–835. 28581484 10.1038/nchembio.2393

[24] S. Ramm , B. Krawczyk , A. Mühlenweg , A. Poch , E. Mösker , R. D. Süssmuth , Angew. Chem. Int. Ed. 2017, 56, 9994–9997; 10.1002/anie.201703488 28715095

[25] E. Matabaro , H. Kaspar , P. Dahlin , D. L. V. Bader , C. E. Murar , F. Staubli , C. M. Field , J. W. Bode , M. Künzler , Sci. Rep. 2021, 11, 3541. 33574430 10.1038/s41598-021-83106-2 PMC7878506

[26] H. Song , N. S. van der Velden , S. L. Shiran , P. Bleiziffer , C. Zach , R. Sieber , A. S. Imani , F. Krausbeck , M. Aebi , M. F. Freeman , et al., Sci. Adv. 2018, 4, eaat2720. 30151425 10.1126/sciadv.aat2720 PMC6108569

[27] C. Ongpipattanakul , S. K. Nair , ACS Chem. Biol. 2018, 13, 2989–2999. 30204409 10.1021/acschembio.8b00668 PMC6340302

[28] H. Song , J. H. Naismith , Curr. Opin. Struct. Biol. 2020, 65, 79–88. 32653730 10.1016/j.sbi.2020.06.004

[29] H. Song , J. R. Fahrig-Kamarauskaitè , E. Matabaro , H. Kaspar , S. L. Shirran , C. Zach , A. Pace , B.-A. Stefanov , J. H. Naismith , M. Künzler , ACS Chem. Biol. 2020, 15, 1901–1912. 32491837 10.1021/acschembio.0c00237 PMC7372559

[30] M. R. Quijano , C. Zach , F. S. Miller , A. R. Lee , A. S. Imani , M. Künzler , M. F. Freeman , J. Am. Chem. Soc. 2019, 141, 9637–9644. 31117659 10.1021/jacs.9b03690

[31] M. Montalbán-López , T. A. Scott , S. Ramesh , I. R. Rahman , A. J. van Heel , J. H. Viel , V. Bandarian , E. Dittmann , O. Genilloud , Y. Goto , et al., Nat. Prod. Rep. 2021, 38, 130–239. 32935693 10.1039/d0np00027b PMC7864896

[32] F. Oldach , R. Al Toma , A. Kuthning , T. Caetano , S. Mendo , N. Budisa , R. D. Süssmuth , Angew. Chem. Int. Ed. 2012, 51, 415–418; 10.1002/anie.201106154 22128014

[33] N. Budisa , Curr. Opin. Biotechnol. 2013, 24, 591–598. 23537814 10.1016/j.copbio.2013.02.026

[34] S. Zobel , S. Boecker , D. Kulke , D. Heimbach , V. Meyer , R. D. Süssmuth , ChemBioChem 2016, 17, 283–287. 26663099 10.1002/cbic.201500649

[35] N. Kakkar , J. G. Perez , W. R. Liu , M. C. Jewett , W. A. van der Donk , ACS Chem. Biol. 2018, 13, 951–957. 29439566 10.1021/acschembio.7b01024 PMC5910287

[36] T. W. Muir , D. Sondhi , P. A. Cole , Proc. Natl. Acad. Sci. USA 1998, 95, 6705–6710. 9618476 10.1073/pnas.95.12.6705 PMC22605

[37] M. Vila-Perelló , Z. Liu , N. H. Shah , J. A. Willis , J. Idoyaga , T. W. Muir , J. Am. Chem. Soc. 2013, 135, 286–292. 23265282 10.1021/ja309126m PMC3544275

[38] H. Iwai , S. Züger , J. Jin , P.-H. Tam , FEBS Lett. 2006, 580, 1853–1858. 16516207 10.1016/j.febslet.2006.02.045

[39] J. Zettler , V. Schütz , H. D. Mootz , FEBS Lett. 2009, 583, 909–914. 19302791 10.1016/j.febslet.2009.02.003

[40] N. H. Shah , G. P. Dann , M. Vila-Perelló , Z. Liu , T. W. Muir , J. Am. Chem. Soc. 2012, 134, 11338–11341. 22734434 10.1021/ja303226x PMC3535263

[41] N. H. Shah , T. W. Muir , Chem. Sci. 2014, 5, 446–461. 24634716 10.1039/C3SC52951G PMC3949740

[42] A. J. Stevens , Z. Z. Brown , N. H. Shah , G. Sekar , D. Cowburn , T. W. Muir , J. Am. Chem. Soc. 2016, 138, 2162–2165. 26854538 10.1021/jacs.5b13528 PMC4894280

[43] A. J. Stevens , G. Sekar , N. H. Shah , A. Z. Mostafavi , D. Cowburn , T. W. Muir , Proc. Natl. Acad. Sci. USA 2017, 114, 8538–8543. 28739907 10.1073/pnas.1701083114 PMC5559002

[44] H. Liang , W. S. Sandberg , T. C. Terwilliger , Proc. Natl. Acad. Sci. USA 1993, 90, 7010–7014. 8346210 10.1073/pnas.90.15.7010 PMC47065

[45] H. L. Zhao , X. Q. Yao , C. Xue , Y. Wang , X. H. Xiong , Z. M. Liu , Protein Expression Purif. 2008, 61, 73–77. 10.1016/j.pep.2008.04.013 18541441

[46] C. Rullán-Lind , R. B. Pietri , M. Vázquez-Cintrón , A. Baerga-Ortiz , Protein Sci. 2018, 27, 969–975. 29520922 10.1002/pro.3404 PMC5916124

